# Recent Advances in Resveratrol Derivatives: Structural Modifications and Biological Activities

**DOI:** 10.3390/molecules30040958

**Published:** 2025-02-19

**Authors:** Xiaohan Liu, Jian Pei, Jiahui Li, Huiyu Zhu, Xiaoyu Zheng, Xingxing Zhang, Banfeng Ruan, Liuzeng Chen

**Affiliations:** School of Biology, Food and Environment, Hefei University, Hefei 230601, China; 15172146475@163.com (X.L.); 13971992017@163.com (J.P.); 18130626743@163.com (J.L.); zhuiyu0713@163.com (H.Z.); m13133517200@163.com (X.Z.); zhangxx@hfuu.edu.cn (X.Z.)

**Keywords:** resveratrol, biological activities, structural modification, structure–activity relationships

## Abstract

Resveratrol, a naturally occurring phenolic stilbene molecule, has been intensively researched for its anti-inflammatory, anticancer, antioxidant, antibacterial, and neuroprotective properties. However, due to its limited absorption and probable hepatotoxicity, it is difficult to employ directly as a medication, limiting its therapeutic applicability. Over the last five years, numerous structural changes in resveratrol have been widely studied, resulting in considerable improvements in pharmacological activity and drug availability. This work reviews the biological activities and structure–activity relationships (SARs) of resveratrol derivatives, with the goal of providing useful insights for the discovery of new resveratrol derivatives.

## 1. Introduction

**Figure 1:**
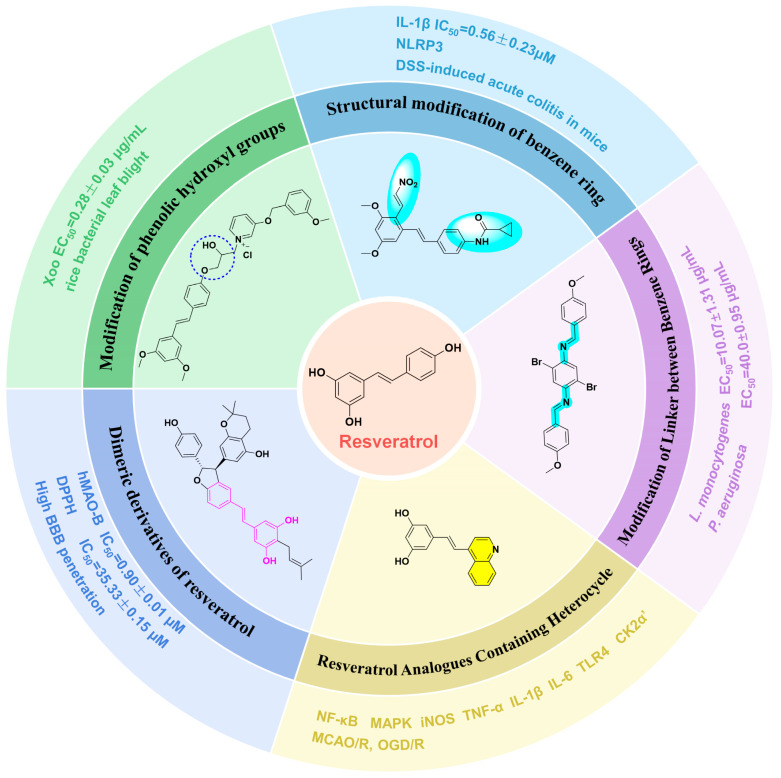
Resveratrol and its structural modification types.

Resveratrol (3,5,4′-trihydroxy-*trans*-stilbene, RSV, Figure 1) is a naturally occurring non-flavonoid polyphenolic compound first isolated from *Eustoma odorata* roots in 1940. Researchers have identified RSV in various plants, such as grapes, mulberries, and peanuts [1,2,3]. The structure of the compound is composed of two hydroxybenzene rings, each with phenolic characteristics, which are bonded together by means of an ethylene connection. In the RSV molecule, the vinyl bridge is responsible for the existence of two stereoisomeric configurations, namely, the *cis-* and *trans-* forms. Among these configurations, the *trans*- form is not only more abundant in plant systems but also demonstrates a higher degree of biological activity [4,5]. RSV acts as a self-protective factor in plants, helping them withstand adversities such as UV light, mechanical damage, and fungal infections. RSV has various biological activities in humans, including anti-inflammatory, anticancer, antioxidant, neuroprotective, and antibacterial properties, among others [6,7,8,9,10,11,12]. However, RSV’s low bioavailability in vivo, poor aqueous solubility, photosensitivity, chemical instability, rapid metabolism, potential hepatotoxicity, potential anticoagulant effect, etc., have combined to limit its development for clinical use and commercialization [13,14,15,16,17].

Medicinal chemists have conducted extensive research to address these issues, including modifications of phenolic hydroxyl groups and benzene rings, alterations to the linkers between benzene rings, the introduction of heterocyclic compounds, and the development of dimeric derivatives of RSV [18,19,20,21,22]. The purpose behind implementing these chemical modifications is to optimize the bioavailability, stability, and solubility of RSV, as well as to refine its targeting capabilities, with the ultimate goal of enhancing its potential use within the realm of pharmaceuticals. In addition, scientists have conducted in vitro cellular, pharmacological, and pharmacokinetic analyses and other experiments on these derivatives to study their mechanisms of action [23,24,25].

The present study provides an overview of advancements in RSV derivatives’ roles in combating inflammation, cancer, oxidative stress, infections, and neurodegeneration over the past half-decade, with a focus on those compounds that exhibit superior benefits. Its goal is to offer a scientific foundation for the creation of new RSV analogs with improved biological activities.

## 2. Modification of Phenolic Hydroxyl Groups

**Figure 2:**
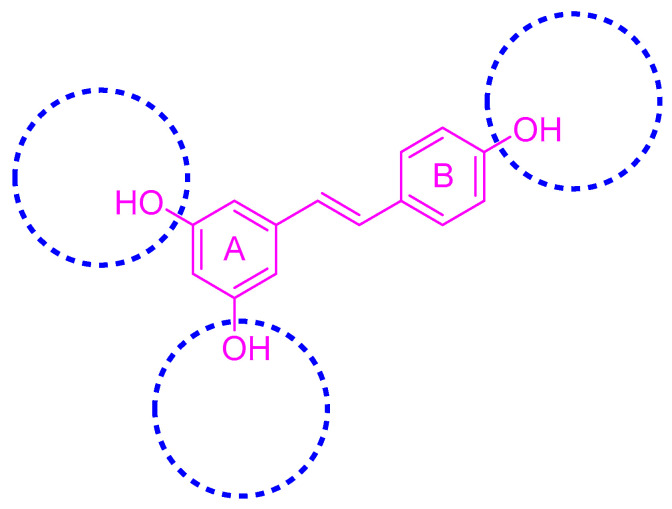
Modification sites of RSV’s phenolic hydroxyl groups.

The phenolic hydroxyl group of RSV is highly susceptible to oxidation (Figure 2), and the modification of one or multiple phenolic hydroxyl groups on RSV with protective groups such as methoxy, ester, amino, benzene sulfonyl, glycoside, etc., can improve its stability, solubility, bioactivity, bioavailability, and targeting [26].

Pterostilbene (PTS, Table 1) is a natural dimethyl derivative of RSV. PTS also possesses anti-inflammatory, antioxidant, antitumor, and neuroprotective activities. In many cases, pterostilbene exhibits significantly higher biological activity than RSV. PTS is distinguished by its low molecular weight and high lipid solubility, which enable it to easily cross the blood–brain barrier (BBB) and be swiftly absorbed and dispersed throughout the body. PTS has appropriate metabolic stability and bioavailability and has no significant toxic effects [27,28,29].

1,2,3-Triazoles are widely used in approved antimicrobial drugs for their increased interaction with targets and increased water solubility. Therefore, Tang et al. (2019) introduced the 1,2,3-triazole structure into the pterostilbene parent nucleus to develop novel agents targeting methicillin-resistant *Staphylococcus aureus* (MRSA) infections. Among them, Compound **1** (Table 1) had the highest anti-MRSA action, with minimum inhibitory concentration (MIC) values of 1.2–2.4 μg/mL and minimum bactericidal concentration (MBC) values of 19.5–39 μg/mL. The ability of Compound 1 to inhibit DNA polymerase was examined, and it was found that Compound 1 could completely inhibit DNA polymerase activity at a concentration of 0.4 μg/mL. This suggests that Compound 1 may inhibit MRSA by acting on DNA polymerase [30].

According to earlier research, 1,3,4-oxadiazole thioether sulfone analogs have good antibacterial action [31]. Peng et al. (2023) introduced this structure on the RSV backbone, and synthesized derivatives mostly showed good antibacterial activity. Compound **2** (Table 1) exhibited median effective concentrations (EC_50s_) of 4.76 ± 0.09 μg/mL against *Xanthomonas oryzae pv. oryzae* (Xoo) and 8.85 ± 1.22 μg/mL against *Xanthomonas oryzae pv. oryzicola* (Xoc). Compound **2** also has the ability to interfere with the normal growth patterns of bacteria by diminishing the creation of biofilms and the production of extracellular polysaccharides (EPSs), and by causing an increase in the permeability of the cell membrane [32].

Among the isopropanol RSV derivatives synthesized by Qi et al. (2022), Compound **3** (Table 1) showed excellent antimicrobial effect, with EC_50s_ of 0.88 ± 0.026 μg/mL for Xoo and 5.71 ± 0.11 μg/mL for *Xanthomonas axonopodis pv. citri* (Xac) [33]. Subsequently, among the novel β-hydroxy pyridinium salt-modified PST derivatives synthesized by Qi et al. (2024), Compound **4** (Table 1) was found to exhibit optimal in vitro antimicrobial activity against Xoo, with an EC_50_ value of 0.28 ± 0.03 μg/mL. Analyses of the antimicrobial mechanism indicated that both Compounds **3** and **4** significantly reduced biofilm formation, EPS biosynthesis, motility, and biofilm assembly, ultimately impairing pathogenicity. Compound **3** also showed a substantial inhibitory effect on pathogenicity, other virulence factors, and bacterial extracellular enzymes. The pot trial revealed that Compound **4** possesses significant control capabilities, exhibiting a therapeutic activity of 71.4% and a protective activity of 72.6%, indicating that it can be employed for efficient in vivo control of rice leaf blight [34].

Tang et al. (2024) synthesized a series of RSV analogs by hybridizing RSV with benzoyl chloride. These compounds significantly inhibited Th2-linked cytokines and chemokines in stimulated keratinocytes through the mitogen-activated protein kinase (MAPK) and c-Jun signaling pathways. They also suppressed Th1/Th17 cytokine and chemokine expression in activated macrophages. Furthermore, these drugs can suppress p-STAT3 and, thus, prevent keratinocytes from interacting with macrophages. Topical administration of Compound **5** (Table 1), which is the most active, improved mice’s atopic dermatitis (AD)-like lesions caused by dinitrochlorobenzene (DNCB) by reducing macrophage recruitment, pro-inflammatory mediators, and epidermal thickness [35].

The BBB presents a significant obstacle to the transportation of drugs to the brain [36]. Combining medicines with glucose or its analogs is one approach to overcoming this barrier and improving brain-targeted medication delivery. These combination chemicals can use glucose transporter protein 1 (GLUT1) to penetrate the BBB, resulting in improved delivery of medication to the brain [37]. Xu et al. (2024) found that the RSV derivatives that they synthesized, incorporating glucose moieties, exhibited neuroprotective properties by mitigating H_2_O_2_-induced neurotoxicity and the production of intracellular reactive oxygen species (ROS). In addition, Compound **6** (Table 1), the most neuroprotective compound, augmented the protective efficacy against cerebral ischemia–reperfusion damage in rats [38].

Belmonte-Reche et al. (2021) created a range of O-silyl RSV derivatives by incorporating modifications like acyl, glucosyl, and carbamoyl groups. These synthesized compounds exhibited excellent neuroprotective and anti-inflammatory properties in vitro. Derivative **7** (Table 1) demonstrated optimal toxicity and neuroprotection, reducing the production of the pro-inflammatory cytokine IL-6 to a greater extent than RSV. Derivative **7** mitigated motor coordination impairment in a 3-nitropropionic acid-induced Huntington’s disease mouse model in an RSV-like manner and markedly lessened disease severity in an experimental autoimmune encephalomyelitis (EAE) multiple sclerosis mouse model [39].

Cebrián et al. (2022) synthesized silicone sulfate and silicone ester RSV derivatives, which exhibited bactericidal activity against Gram-positive bacteria comparable to that of conventional antibiotics. These compounds act by increasing membrane permeability and inducing membrane hyperpolarization, which leads to impairment of the cellular respiratory chain and ATP synthesis, ultimately leading to bacterial death. Compounds **8** and **9** (Table 1), recognized as the most effective, demonstrated activity against all evaluated Gram-positive bacterial strains. Their MICs spanned from 6.6 to 64 μM for Compound **8** and from 4 to 21.3 μM for Compound **9**. In addition, these derivatives synergistically interacted with conventional antibiotics such as aminoglycosides and polymyxin B. Although these derivatives showed significant antibacterial effects on Gram-positive bacteria, they were less effective against Gram-negative bacteria, due to the fact that the outer membrane acts as a barrier to the penetration of these compounds into the inner membrane [40].

Cebrián et al. (2023) then synthesized a variety of aminoalkyl RSV derivatives using the nature of cationic amphiphilic antimicrobial peptides. Derivative **10** (Table 1) exhibited potent antibacterial properties against various anaerobic bacteria, with MICs varying from 13.3 to 64 μM for Gram-negative strains and from 3.3 to 36.7 μM for Gram-positive strains. Although it has some cytotoxicity, it has lower toxicity and hemolytic activity compared to other tested derivatives. It was found that these derivatives exert their antimicrobial effects mainly by interfering with the integrity of the bacterial membrane [41].

Szczepańska et al. (2023) synthesized lipidated RSV derivatives that exhibited superior pharmacological properties and bioavailability compared to other phenolic compounds. Notably, a mixture of the mono-RSV-oleic acid (OA) derivatives, **11** and **12**, showed the strongest anticancer effects against lung cancer cells (A549), colorectal cancer cells (HT29), and pancreatic cancer cells (BxPC3), with human fibroblasts (BJ) as a control group. The compound increased apoptosis in tumor cells by altering the cystatinase activity of pro-apoptotic pathways (p21, p53, and Bax). Regarding antioxidant properties, the mixture notably elevated the expression of the antioxidant genes *SOD1* (superoxide dismutase 1) and *SOD2* in normal BJ cells, thus shielding these cells from heightened oxidative stress caused by excessive ROS accumulation. These findings imply that increasing lipophilic properties can boost the compound’s biological efficacy [42].

**Table 1:**
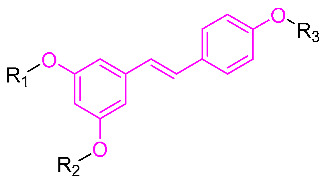
Molecular structure of Compounds **1**–**12** and their biological activities.

Compound	R_1_	R_2_	R_3_	Activity	Molecular Mechanism	In Vivo Model	Ref.
PTS	-CH_3_	-CH_3_	-H	- ^1^	-	-	-
**1**	-CH_3_	-CH_3_	** 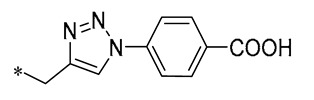 **	MRSA(MIC = 1.2–2.4 μg/mL, MBC = 19.5–39 μg/mL)	Inhibiting DNA polymerase	-	[30]
**2**	-CH_3_	-CH_3_	** 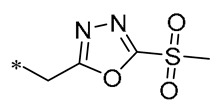 **	Xoo EC_50_ = 4.76 ± 0.09 μg/mL Xoc EC_50_ = 8.85 ± 1.22 μg/mL	Suppressing the formation of bacterial biofilms Suppressing the production of EPS	Rice bacterial leaf blight Rice bacterial leaf streak	[32]
**3**	-CH_3_	-CH_3_	** 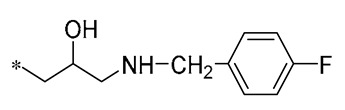 **	Xoo EC_50_ = 0.88 ± 0.026 μg/mL Xac EC_50_ = 5.71 ± 0.11 μg/mL	Suppressing the formation of bacterial biofilms Suppressing the production of EPS	Rice bacterial leaf blight Citrus canker	[33]
**4**	-CH_3_	-CH_3_	** 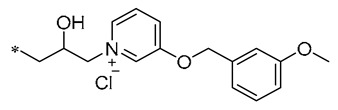 **	Xoo EC_50_ = 0.28 ± 0.03 μg/mL	Suppressing the formation of bacterial biofilms Suppressing the production of EPS	Rice bacterial leaf blight	[34]
**5**	-CH_3_	-CH_3_	** 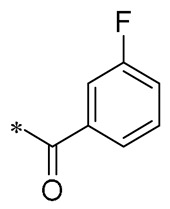 **	Inhibition of Th2, Th1/Th17 expression	Impact on MAPK and c-Jun signaling pathways	DNCB-induced AD-like mice	[35]
**6**	** 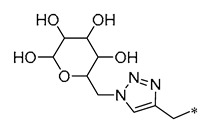 **	-H	-H	Neuroprotection	Mitigating H_2_O_2_-induced neurotoxicity and the production of intracellular ROS	Cerebral ischemia–reperfusion model	[38]
**7**	-TIPS	--TIPS	-β-Glc-Oct	Reducing the production of IL-6	-	EAE multiple sclerosis mouse model	[39]
**8**	-SO_3_^-^	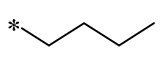	-SO_3_^-^	Anaerobic Gram-positive bacteria (MIC = 6.6–64 μM)	Increasing membrane permeability Inducing membrane hyperpolarization	-	[40]
**9**	-H	-H	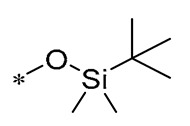	Anaerobic Gram-positive bacteria (MIC = 4–21.3 μM)	Increasing membrane permeability Inducing membrane hyperpolarization	-	[40]
**10**	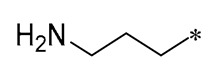	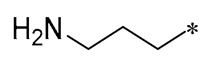	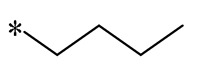	Gram-negative (MIC = 13.3–64 μM) Gram-positive (MIC = 3.3–36.7 μM)	Suppressing the formation of bacterial biofilms	-	[41]
**11**	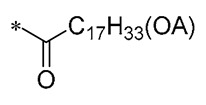	-H	-H	Against A549, BxPC3, and HT29 cell lines	Altered cystatin activity of pro-apoptotic pathways (p21, p53, and Bax) Increased expression of SOD1 and SOD2	-	[42]
**12**	-H	-H	** 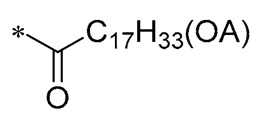 **	Against A549, BxPC3, and HT29 cell lines	Altered cystatin activity of pro-apoptotic pathways (p21, p53, and Bax) Increased expression of SOD1 and SOD2	-	[42]

^1^ Not explicitly stated in the literature.

## 3. Structural Modification of Benzene Ring

**Figure 3:**
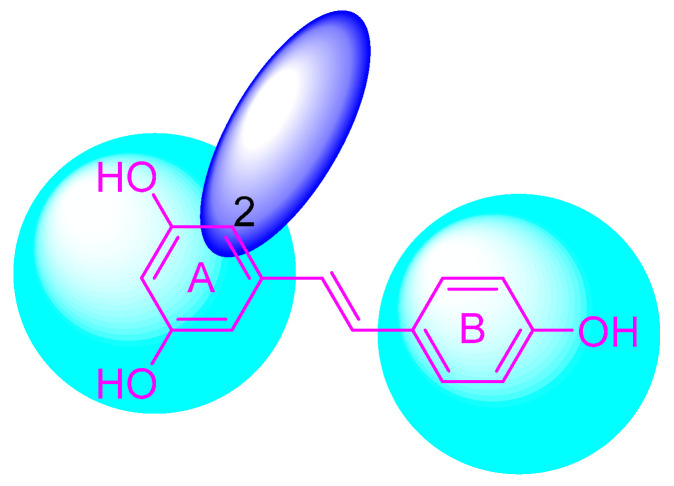
Modification sites on the benzene ring of RSV.

The structural modification on the benzene ring of RSV mainly focuses on the No. 2 position on ring A, such as the introduction of curcumin, chalcone, nitrovinyl, and other structures at the No. 2 position (Figure 3). This can improve its anti-inflammatory, anticancer, antimicrobial, antioxidant, and neuroprotective bioactivities, as well as enhancing the targeting properties.

In prior research, Wu et al. (2019) discovered that a mixture of RSV and curcumin monocarbonyl (**13** and **14**, Table 2) exhibited superior anti-inflammatory activity and low toxicity. Compound **13** was effective in preventing lipopolysaccharide (LPS)-induced acute lung injury in mice [43]. In subsequent research, Compound **14** demonstrated a significant reduction in lipid accumulation, liver damage, hepatic inflammation, and fibrosis caused by a high-fat diet (HFD) in a murine model. Specifically, Compound **14** notably decreased the transcription of inflammatory mediators (such as TNF-α, IL-6, IL-1β, and COX-2) and the expression of adhesion molecules (including ICAM, VCAM-1, and MCP-1) stimulated by palmitic acid (PA). Its anti-inflammatory action may be mediated by the ERK signaling pathway. The treatment involving Compound **14** has been shown to lessen body weight gain, hepatic fat accumulation, liver damage, and pathological changes in mice subjected to a high-fat diet. Compound **14** mitigated hepatic inflammation triggered by a high-fat diet by reducing the mRNA levels of inflammatory cytokines and the number of F4/80-positive cells in the liver. Additionally, it downregulated the expression of fibrotic genes like α-SMA, COL-I, COL-IV, and TGF-β, and it decelerated collagen accumulation in livers affected by an HFD. These findings indicate that **14** could be employed as a protective agent against NAFID [44]. Zhang et al. (2019) also found that **14** attenuated HFD-induced cardiac and renal inflammation and injury [45].

Ma et al. (2019) synthesized a novel piperazine-substituted chalcone RSV derivative (**15**, Table 2), which showed good results in inhibiting NO production, with an IC_50_ = 4.13 ± 0.07 μM. In addition, it demonstrated potent antiproliferative effects against three human tumor cell lines (Hela, A549, and SGC7901), with IC_50s_ of 4.042 ± 0.16, 27.72 ± 1.45, and 3.93 ± 0.37 μM, respectively [46].

Chen et al. (2019) developed a number of novel flavonoid RSV compounds and discovered that including hydroxyl groups within the flavonoid structure improved the efficacy of suppressing NO release. Compound **16** (Table 2) proved to be the most potent in diminishing the intracellular production of NO, IL-6, and TNF-α, with respective IC_50_ values of 1.35, 1.12, and 1.92 μM. Preliminary mechanism studies indicated that it could inhibit the expression of TLR4 protein, resulting in activation of the NF-κB cell signaling pathway. Additionally, it significantly mitigated LPS-induced pulmonary inflammation and acute lung damage in mice [47].

Tang et al. (2021) created a series of RSV derivatives of chalcone cinnamate that were found to have anticancer activity against oral cancer cell lines. Derivative **17** (Table 2) demonstrated superior anticancer potency, registering IC_50_ values of 16.38 ± 0.10 µM against OECM-1 and 18.06 ± 0.05 µM against HSC-3 cell lines. The data indicated that this derivative significantly curtailed cellular proliferation and induced a G2/M cell-cycle arrest through the regulation of p21, cyclin B1, and cyclin A2. Ultimately, it enhanced apoptotic activity by reducing Bcl-2 and survival protein levels, and by increasing the cleavage of PARP and caspase-3 at elevated concentrations [48].

Fang et al. (2021) synthesized a series of new RSV derivatives by introducing an indone structure to the RSV backbone. The most potent derivative, **18** (Table 2), effectively lowered the LPS-stimulated mRNA expression of IL-1β, TNF-α, iNOS, and COX-2. The anti-inflammatory activity of Compound **18** is believed to be facilitated by the suppression of the NF-κB and MAPKs signaling pathways. Furthermore, in vivo experiments demonstrated that Compound **18** mitigated LPS-stimulated sepsis and decreased multi-organ toxicity in C57BL/6J mice [49].

Park et al. (2023) created a variety of RSV/pentadienone hybrid compounds. Compound **19** (Table 2) demonstrated the most potent suppressive impact on colony formation in MDA-MB-231 breast cancer cells, achieving a GI_50_ of 0.35 µM. This compound disrupted the stability of microtubule proteins and impeded the cell cycle’s advancement at the G2 phase. Additionally, computational molecular docking studies indicated that Compound **19**’s binding affinity resembled that of colchicine [50].

Yang et al. (2023) synthesized Derivative **20** (Table 2), which has antioxidant and anti-inflammatory activities, by introducing chalcone and dihydropyrazole structures into the RSV parent nucleus. Derivative **20** diminishes the formation of ROS in RAW264.7 and H9C2 cells by enhancing the expression of antioxidant enzymes, including Nrf2, SOD1, catalase (CAT), and glutathione peroxidase 1 (GPX1). Furthermore, it lessens inflammation provoked by LPS or adriamycin by suppressing the NF-κB signaling pathway and reducing the expression of inflammatory factors. In a mouse model, Derivative 20 was shown to attenuate adriamycin-induced heart failure [51]. Wei et al. (2023) investigated the effects of Derivative **21** (Table 2) on inflammation regulation and vascular calcification. They found that this compound reduced the expression of phosphorylated extracellular signal-regulated kinase 1/2 (p-ERK1/2) and β-binding proteins, along with the inflammatory cytokines IL-1β, TNF-α, and IL-6, in both in vivo and in vitro settings. Derivative **21** substantially lessened calcium and lipid accumulation in the aorta and aortic root, and it markedly slowed the progression of atherosclerosis and intimal calcification in a murine model of the condition [52].

Chen et al. (2024) developed innovative RSV derivatives by integrating a thiazole molecule into the RSV framework using a pharmacophore interaction approach. Derivatives **22** and **23** (Table 2) exhibited superior activity, with IC_50_ values against NO of 0.7 ± 0.15 μM and 0.6 ± 0.12 μM, respectively. Both **22** and **23** were found to decrease NO and PGE2 production by suppressing the expression of iNOS and COX-2. They mitigated inflammation by reducing the phosphorylation of P65 and IκB in the NF-κB pathway and p38, ERK, and JNK in the MAPKs system. In vivo mouse studies have shown that Derivatives **22** and **23** have beneficial alleviating effects on DSS-induced acute colitis, including a decrease in body weight loss and disease index, and they exhibit a favorable safety profile in acute toxicity assessments [53,54].

Most of the novel RSV derivatives synthesized by Peng et al. (2024) containing 1,3,4-oxadiazole and amide groups exhibited excellent antimicrobial activity against Xoc and Xoo. In particular, Derivative **24** (Table 2) showed EC_50_ values of 4.2 ± 1.2 μg/mL and 5.0 ± 0.5 μg/mL, respectively. Preliminary mechanistic studies revealed that **24** could greatly inhibit biofilm formation, EPS production, and extracellular enzyme synthesis while also disrupting bacterial cell surface shape and lowering bacterial pathogenicity. Moreover, Derivative **24** enhanced the functionality of defense-related enzymes and modulated the expression of multiple genes associated with pathogen responses, including plant–pathogen interactions, the MAPK signaling pathway, and the biosynthesis of phenylpropanoids, which, in turn, bolstered rice’s resistance to bacterial leaf streak disease [55].

Chen et al. (2021) constructed a novel high-intensity screening model for screening small inhibitors targeting NLRP3 and identified PTS as an active scaffold. Subsequently, 50 derivatives of pterostilbene were synthesized. Compound **25** (Table 2) had the highest inhibition rate (IR) of 73.09% at 10 μM and excellent potency against IL-1β (IC_50_ = 0.56 ± 0.23 μM). Subsequent investigations indicated that Compound **25** modulated the assembly of NLRP3 inflammasomes by directly impacting NLRP3. In vivo bioactivity assays demonstrated that this chemical significantly alleviated DSS-triggered colitis in mice. Additionally, it was determined that the nitrovinyl substitution at position 2 is an essential active motif of PTS for NLRP3 targeting [56].

On this basis, Zhang et al. (2022, 2024) and Ruan et al. (2023) developed a variety of PTS derivatives, among which Compounds **26**, **27**, and **28** (Table 2) all showed good inhibitory effects on IL-1β and exhibited good safety and excellent anti-inflammatory activities in cellular assays. In addition, Compound **27** is capable of significantly lowering the release of the inflammatory cytokine IL-1β without triggering the NF-κB pathway, indicating that it possesses a distinct anti-inflammatory mode of action. Compounds **26**, **27**, and **28** all act directly on the NLRP3 protein, altering inflammasome assembly, decreasing NLRP3 inflammasome activation, and preventing ASC protein oligomerization. Furthermore, they all demonstrated significant therapeutic efficacy in a mouse model of DSS-induced colitis while maintaining good metabolic stability in liver microsomes, and they are all predicted to be novel medications for the treatment of Inflammatory Bowel Disease (IBD) [57,58,59].

Among the benzoylhydrazine RSV derivatives synthesized by Lu et al. (2024), Derivative **29** (Table 2) was found to have excellent scavenging of free radicals and reduction of iron ions. In the assessment of free radical scavenging abilities, Derivative **29** showed varying potencies, with IC_50_ values of 69.9 ± 1.8 μM for DPPH, 13 ± 0.2 μM for ABTS, and 1.1 ± 0.05 μM for FRAP, indicating its antioxidant potential across different assays. Administered at 5 μM, Derivative **29** efficiently lowered ROS generation in RAW 264.7 cells challenged with H_2_O_2_, showing similar effectiveness to RSV at a 10 μM dose. It also displayed superior suppression of NO in LPS-activated RAW 264.7 cells. Additionally, Derivative **29** fully engaged the Nrf2 signaling pathway and boosted heme oxygenase-1 (HO-1) levels under H_2_O_2_-induced oxidative conditions. Derivative **29** lessened cell damage and apoptosis by curbing the expression of pro-apoptotic proteins like cystathionase 3 and PARP. Additionally, it restrained the activation of the NF-κB, p65/iNOS, and MAPKs pathways, which contributed to its anti-inflammatory properties [60]. Lamya et al. (2024) synthesized benzoylhydrazine derivatives, specifically Derivative **30** (Table 2), which exhibited optimal cytotoxic effects, with IC_50_ values of 24.62 μM and 70.92 μM against the breast cancer cell lines ICF-7 and T47D, respectively [61].

Among the synthesized RSV analogs crafted by Subramanian et al. (2024), Derivative **31** (Table 2) manifested the most potent inhibition against acetylcholinesterase (AChE) and butyrylcholinesterase (BChE), registering IC_50_ values of 40.60 μg/mL and 40.18 μg/mL, respectively. In the DPPH radical scavenging test, Derivative **31** exhibited robust antioxidant properties, achieving an IC_50_ value of 46.22 μg/mL. It efficiently mitigated oxidative stress induced by β-amyloid in the DCFDA ROS assay, showing antioxidant efficacy on par with that of established medications [62].

**Table 2:**
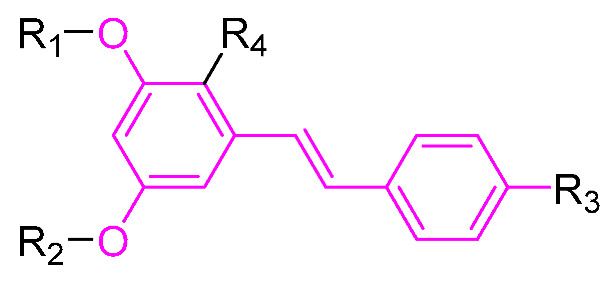
Molecular structure of Compounds **13**–**31** and their biological activities.

Compound	R_1_	R_2_	R_3_	R_4_	Activity	Mechanism	In Vivo Model	Ref.
**13**	-CH_3_	-CH_3_	-OCH_3_	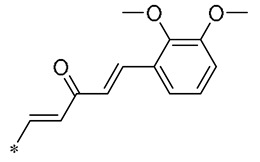	Inhibited the LPS-induced production of IL-6 and TNF-a	Decreased LPS-induced TNF-a, IL-6, IL12, and IL-33 mRNA expression	LPS-induced acute lung injury in the in vivo mouse model	[43]
**14**	-CH_3_	-CH_3_	-OCH_3_	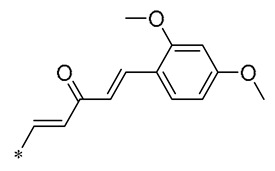	Decreased the transcription of inflammatory mediators (such as TNF-α, IL-6, IL-1β, and COX-2) and the expression of adhesion molecules (including ICAM, VCAM-1, and MCP-1) stimulated by PA	Affects the ERK signaling pathway	HFD-induced NAFLD HFD-induced heart and kidney injury	[43,44,45]
**15**	-CH_3_	-CH_3_	-OCH_3_	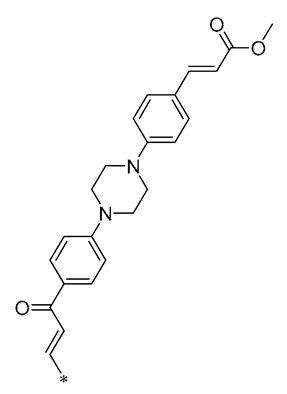	NO IC_50_ = 4.13 ± 0.07 μM. Hela IC_50_ = 4.042 ± 0.16 μM. A549 IC_50_ = 27.72 ± 1.45 μM. SGC7901 IC_50_ = 3.93 ± 0.37 μM	- ^1^	-	[46]
**16**	-CH_3_	-CH_3_	-OCH_3_	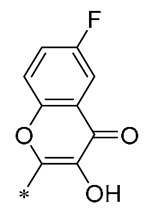	NO IC_50_ = 1.35 μM IL-6 IC_50_ = 1.12 μM TNF-α IC_50_ = 1.92 μM	Inhibits the expression of TLR4 protein, resulting in activation of the NF-κB cell signaling pathway	LPS-induced acute lung injury	[47]
**17**	-CH_3_	-CH_3_	-OH	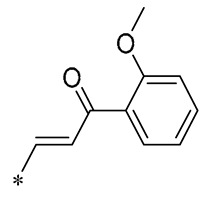	OECM-1 IC_50_ = 16.38 µM HSC-3 IC_50_ = 18.06 µM	Inhibits cell proliferation and induces G2/M cell-cycle arrest by regulating p21, cyclin B1, and cyclin A2 Reducing Bcl-2 and survival protein levels, and increasing PARP and caspase-3 cleavage at higher concentrations	-	[48]
**18**	-CH_3_	-CH_3_	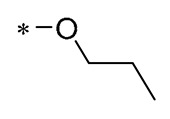	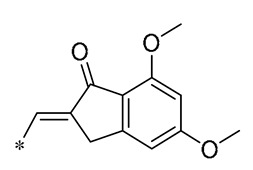	Inhibited the LPS-induced production of IL-1β, TNF-α, iNOS, and COX-2	Inhibition of NF-κB and MAPKs signaling pathways	LPS-induced sepsis in C57BL/6J mice and reduced multi-organ toxicity	[49]
**19**	-CH_3_	-CH_3_	-OCH_3_	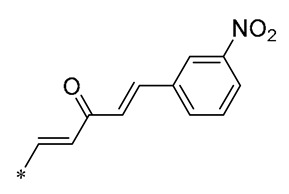	MDA-MB-231 GI_50_ = 0.35 µM	Disrupted the stability of microtubule proteins	-	[50]
**20**	-CH_3_	-CH_3_	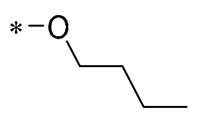	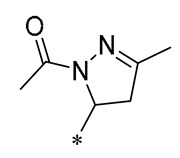	Increased expression of Nrf2, SOD1, CAT, and GPX1 Decreased ROS generation	Inhibiting NF-κB signaling	DOX-induced heart failure	[51]
**21**	-CH_3_	-CH_3_	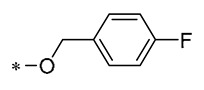	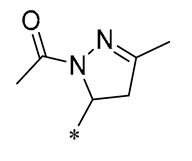	Reduced IL-6, L-1β, and TNF-α expression	Blocks the ERK1/2 signaling pathway	Medial calcification induced by nicotine and VD3	[52]
**22**	-CH_3_	-CH_3_	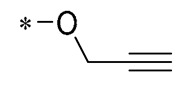	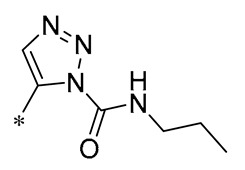	NO IC_50_= 0.7 ± 0.15 μM	Inhibition of NF-κB and MAPKs signaling pathways	DSS-induced acute colitis in mice	[53]
**23**	-CH_3_	-CH_3_	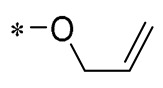	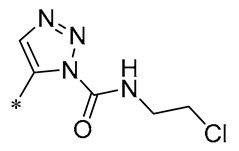	NO IC_50_= 0.6 ± 0.12 μM	Inhibition of NF-κB and MAPKs signaling pathways	DSS-induced acute colitis in mice	[54]
**24**	-CH_3_	-CH_3_	-OCH_3_	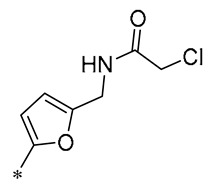	Xoo EC_50_ = 4.2 ± 1.2 μg/mL Xoc EC_50_ = 5.0 ± 0.5 μg/mL	Suppressing the formation of bacterial biofilms	Rice bacterial leaf blight Rice bacterial leaf streak	[55]
**25**	-CH_3_	-CH_3_	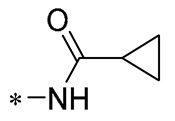	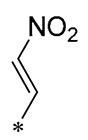	IL-1β IC_50_ = 0.56 ± 0.23 μM	Influences the assembly of NLRP3 inflammatory vesicles by targeting NLRP3.	DSS-induced acute colitis in mice.	[56]
**26**	-CH_3_	-CH_3_	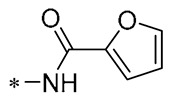	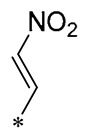	IL-1β IC_50_ = 1.23 ± 0.51 μM	Influences the assembly of NLRP3 inflammatory vesicles by targeting NLRP3	DSS-induced acute colitis in mice	[58]
**27**	-CH_3_	-CH_3_	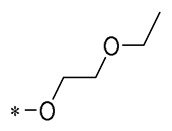	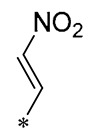	IL-1β IC_50_ = 2.41 μM	Influences the assembly of NLRP3 inflammatory vesicles by targeting NLRP3	DSS-induced acute colitis in mice	[59]
**28**	-CH_3_	-CH_3_	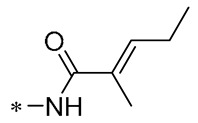	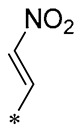	IL-1β IC_50_ = 2.99 μM	Influences the assembly of NLRP3 inflammatory vesicles by targeting NLRP3	DSS-induced acute colitis in mice	[57]
**29**	-H	-H	-H	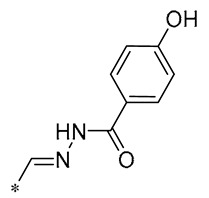	DPPH IC_50_ = 69.9 ± 1.8 μM ABTS IC_50_ = 13 ± 0.2 μM FRAP IC_50_ = 1.1 ± 0.05 μM	Inhibition of NF-κB, p65/iNOS, and MAPKs pathway activation	-	[60]
**30**	-H	-H	-H	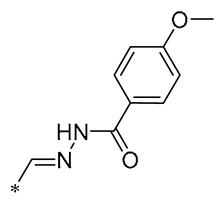	ICF-7 IC_50_ = 24.62 ± 4.70 μM T47D IC_50_ = 70.92 ± 21.28 μM	-	-	[61]
**31**	-CH_3_	-CH_3_	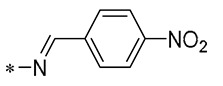	-H	AChE IC_50_ = 40.60 μg/mL BChE IC_50_ = 40.18 μg/mL	-	-	[62]

^1^ Not explicitly stated in the literature.

**Figure 4:**
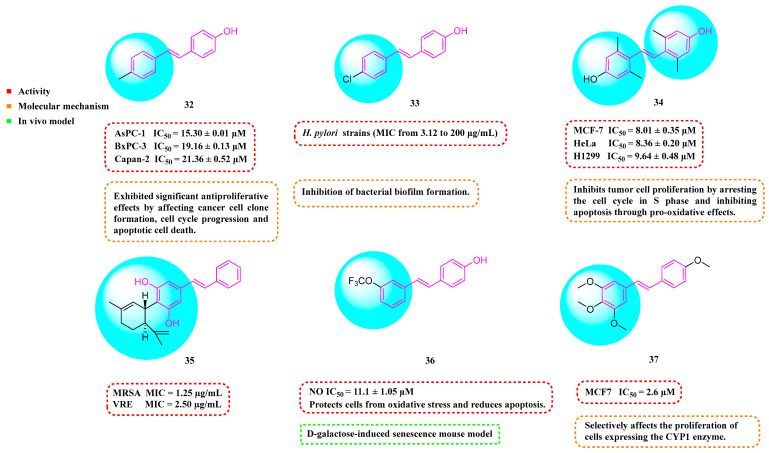
Molecular structure of compounds **32**–**37** and their biological activities.

Barbara et al. (2019) synthesized a variety of simplified RSV analogs. The cytotoxic effects of these compounds were evaluated in three pancreatic cancer cell lines with unique genetic signatures: AsPC-1, BxPC-3, and Capan-2. Compound **32** (Figure 4) showed the best cytotoxic activity [63]. Florio et al. (2023) also found that **32** significantly reduced the expression of CD133 and EpCAM markers on the surface of pancreatic cancer (PC) stem cells and restricted PC cell cloning by further investigation of **32**. In AsPC-1 cancer cells, **32** was more effective than RSV at activating the DNA damage marker H2AX and promoting altered expression of cell-cycle regulatory proteins, which interfered with AsPC-1 cancer cells’ biological processes and effectively promoted apoptosis [64].

Fermo et al. (2020) found that both RSV and their newly synthesized RSV derivatives showed antimicrobial effects against drug-resistant *H. pylori*. Among them, Compound **33** (Figure 4) showed stronger antibacterial activity relative to RSV (MIC from 3.12 to 200 μg/mL). Compound **33** was shown to inhibit the formation of bacterial biofilms and reduce the clustering of H. pylori on soft agar. Additionally, Compound **33** was non-toxic to greater wax borer larvae and provided protection against H. pylori infection [65].

Xin et al. (2020) synthesized a series of hydroxy stilbene-methylated RSV analogs, among which Compound **34** (Figure 4) showed the strongest cytotoxicity against tumor cells (MCF-7, HeLa, and H1299), with IC_50s_ of 8.01 ± 0.35, 8.36 ± 0.20, and 9.64 ± 0.48 μM, respectively. It was shown that Compound **34** inhibited the proliferation of tumor cells through the blockade of the S-phase cell cycle and inhibited apoptosis by promoting oxidation [66].

Kumarihamy et al. (2022) synthesized a series of RSV analogs. Of these analogs, **35** (Figure 4) showed significant antimicrobial activity in studies, with an MIC of 1.25 µg/mL against MRSA. The MIC against vancomycin-resistant *Enterococcus faecium* and *E. faecalis* (VRE) was 2.50 µg/mL [67].

Of the RSV derivatives synthesized by Liang et al. (2023), **36** showed inhibition of NO production (IC_50_ = 11.1 ± 1.05 μM), suppression of oxidative toxicity, reduction in ROS accumulation, and apoptotic activity in an in vitro oxidative stress cell model. Within a mouse model of senescence triggered by D-galactose, Derivative **36** successfully reversed liver and kidney impairment, shielded the serum, brain, and liver from oxidative stress, and bolstered the immune response in the spleen. Furthermore, Derivative **36** mitigates brain aging by safeguarding cells against oxidative stress and diminishing apoptosis [68].

Ruparelia et al. (2024) synthesized a series of methyl ether analogs of RSV. These RSV analogs were significantly more potent on cytochrome P450 1 (CYP1)-expressing breast tumor cell lines than on cell lines without CYP1 activity. Of these, Analog **37** (Figure 4) had the strongest ability to inhibit cancer cell proliferation. The compound exhibited mild-to-moderate cytotoxic effects, with an IC_50_ value of 2.6 μM against MCF7 cells. However, it formed highly toxic metabolites during the CYP1-mediated dealkylation reaction, showing an IC_50_ of 0.12 μM specifically for MCF7 cells activated by 2,3,7,8-tetrachlorodibenzo-p-dioxin (TCDD). Consequently, Compound **37** selectively targets cell proliferation in cells that express the CYP1 enzyme [69].

## 4. Modification of Linkers Between Benzene Rings

The double bond (C=C) in RSV acts as a linker between two aromatic rings, which greatly influences the biological activity of polyphenols. Consequently, certain scientists have engineered RSV derivatives by substituting the C=C bond with either a C=N or N=N bond through a bioisosteric replacement strategy. Moreover, inflexible moieties like the C=C double bond can be swapped for more pliable groups such as amides, amines, and single carbon–carbon bonds to enhance the biological efficacy of RSV derivatives [70,71,72,73,74,75].

**Figure 5:**
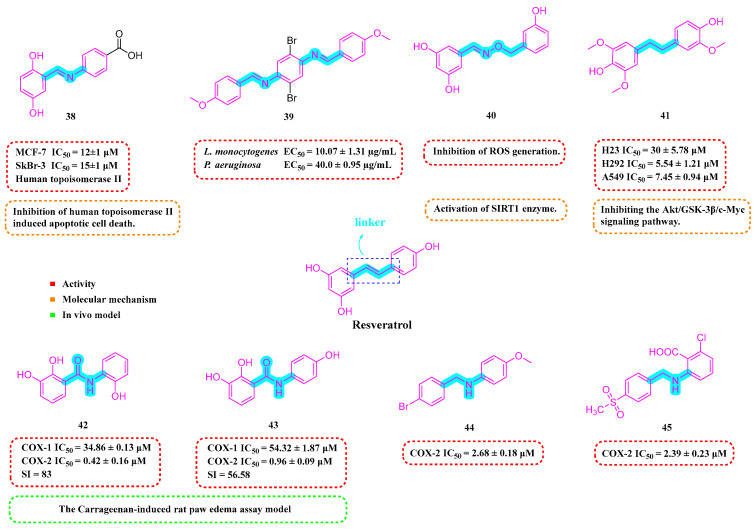
Molecular structure of compounds **38**–**45** and their biological activities.

Iacopetta et al. (2020) developed a range of RSV imino derivatives. Their findings indicated that Derivative **38** (Figure 5) demonstrated the most effective antiproliferative properties in two breast cancer cell lines, MCF-7 and SkBr-3, with IC_50_ values of 12 ± 1 μM and 15 ± 1 μM, respectively. Moreover, Derivative **38** displayed enhanced bioavailability compared to the parent RSV in both gastric and small-intestinal conditions, with a 35% higher overall value relative to RSV. In addition, **38** could induce apoptotic cell death by inhibiting human topoisomerase II [76].

The most active of the Schiff base hybrid resveratrol derivatives synthesized by Sánchez-González et al. (2022) was Compound **39** (Figure 5), which showed good performance in antimicrobial activity tests against *Listeria monocytogenes* (LM), EC_50_ = 10.07 ± 1.31 µg/mL. Furthermore, Compound **39** demonstrated potent antimicrobial effects against *Pseudomonas aeruginosa*, with an EC_50_ value of 40.0 ± 0.95 µg/mL [77].

Ciccone et al. (2022) developed Compound **40** (Figure 5), which demonstrated superior antioxidant capabilities compared to RSV in the ABTS radical scavenging test. Compound **40** was able to safeguard the viability of human umbilical vein endothelial cells (HUVECs) by modulating SIRT1 and could counteract the rise in intracellular ROS triggered by H_2_O_2_ [78].

Thongsom et al. (2023) synthesized RSV Analog **41** (Figure 5), which demonstrated greater efficacy in suppressing the viability of lung cancer cell lines (H23, H292, and A549) than RSV itself. Additionally, Compound **41** can increase intracellular ROS levels and induce oxidative stress, leading to apoptosis in cancer cells. Compared to RSV, **41** exhibits a more potent capability to curb the CSC-like characteristics of lung cancer cells, achieving this by inhibiting the Akt/GSK-3β/c-Myc signaling pathway [79].

Xin et al. (2023) evaluated the inhibitory activity of synthesized RSV amide derivatives against COX-1/COX-2. The findings revealed that Compounds **42** and **43** (Figure 5) demonstrated potent inhibitory effects (IC_50s_ of 0.42 ± 0.16 μM and 0.96 ± 0.09 μM, respectively) and marked selectivity (selectivity indices (SIs) of 83 and 56.58, respectively) for COX-2, implying that they inhibited COX-2 much more than COX-1. The compounds’ in vivo anti-inflammatory properties were further evaluated using a carrageenan-induced rat paw edema assay, with the research indicating that Compound 41 demonstrated substantial anti-inflammatory activity, achieving 45.95% inhibition of edema at three oral dosages of 50 mg/kg [80].

Wu et al. (2024) developed a range of N-substituted structurally modified RSV derivatives and assessed their anti-inflammatory and antitumor capabilities in vitro. Compounds **44** and **45** (Figure 5) strongly suppressed NO generation at 10 μm concentrations, with concentration-dependent inhibition. In addition, Compounds **44** and **45** demonstrated effective COX-2 inhibition (IC_50s_ of 2.68 ± 0.18 and 2.39 ± 0.23 μM, respectively). Compound **45** inhibited various tumor cell lines while inducing apoptosis in MCF-7 breast cancer cells [81].

## 5. Resveratrol Analogs Containing Heterocycle

**Figure 6:**
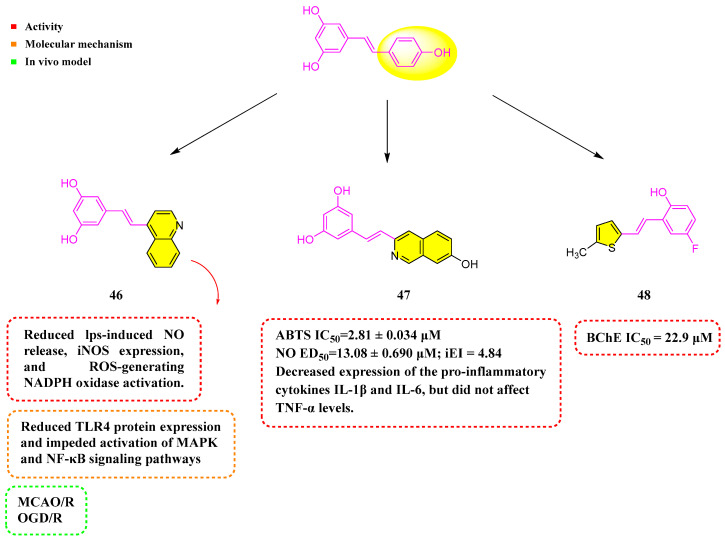
Molecular structure of compounds **46**–**48** and their biological activities.

Hou et al. (2019) synthesized a quinolinyl-substituted RSV analog, **46** (Figure 6). It was shown that **46** reduced LPS-induced NO release, iNOS expression, ROS generation, and NADPH oxidase activation. Moreover, Compound **46** reduced the expression of TLR4 protein and hindered the activation of the MAPK and NF-κB signaling pathways triggered by LPS. In the rat model of middle cerebral artery occlusion–reperfusion (MCAO/R), Compound 45 decreased the volume of infarction and neurological deficits, improved performance on the rotarod test, and lessened the frequency of rightward turns. In the oxygen–glucose deprivation and reperfusion (OGD/R) model using primary microglial cells, Compound **46** mitigated oxidative stress and mitochondrial damage by promoting mitochondrial autophagy in SH-SY5Y cells subjected to OGD/R. Additionally, Compound **46** could lower the mRNA expression of the pro-inflammatory cytokines IL-6, TNF-α, and IL-1β, potentially alleviating neuroinflammation. Significantly, TLR4 knockdown dramatically improved **46**’s anti-inflammatory activities, whereas TLR4 overexpression hindered them. Compound **46** promoted mitophagy by interacting with CK2α’, reducing damage caused by acute ischemic strokes. These insights reveal potential mechanisms through which Compound **46** could treat ischemic stroke (IS) [82,83,84].

Nam et al. (2021) prepared a number of analogs by chemical synthesis, including structures containing naphthalene and its bioelectronic isomers. ABTS experiments showed that all compounds possessed better antioxidant activity than RSV. Among them, Analog **47** (Figure 6) showed the strongest activity, with IC_50_ = 2.81 ± 0.034 µM. Moreover, Compound 46 decreased the expression of the pro-inflammatory cytokines IL-1β and IL-6 in RAW 264.7 cells when stimulated by LPS, yet it did not impact TNF-α levels [85].

Mlakić et al. (2024) synthesized 14 RSV analogs using heterocyclic-containing triphenylphosphine salts and different benzaldehydes via the Wittig reaction. The compounds underwent evaluation for their potency to suppress AChE and BChE, in addition to assessing their antioxidant capabilities. Among the heteroaromatic RSV derivatives, they demonstrated greater efficacy in curbing BChE activity. Specifically, Analog **48** (Figure 6) exhibited the most pronounced inhibitory effect on BChE, registering an IC_50_ of 22.9 μM. In addition, these compounds showed stronger antioxidant activity than standard RSV [86].

## 6. Dimeric Derivatives of Resveratrol

Both resveratrol polymers and RSV are naturally occurring polyphenolic compounds that are present in various plants. These resveratrol polymers have garnered attention due to their wide range of biological activities, such as antioxidant, anti-inflammatory, antibacterial, anti-neoplastic, and neuroprotective capabilities. Predominantly, in their natural state, resveratrol polymers are found as oligomeric forms, with certain oligomeric polymers exhibiting significantly higher activity levels compared to RSV [87,88,89].

**Figure 7:**
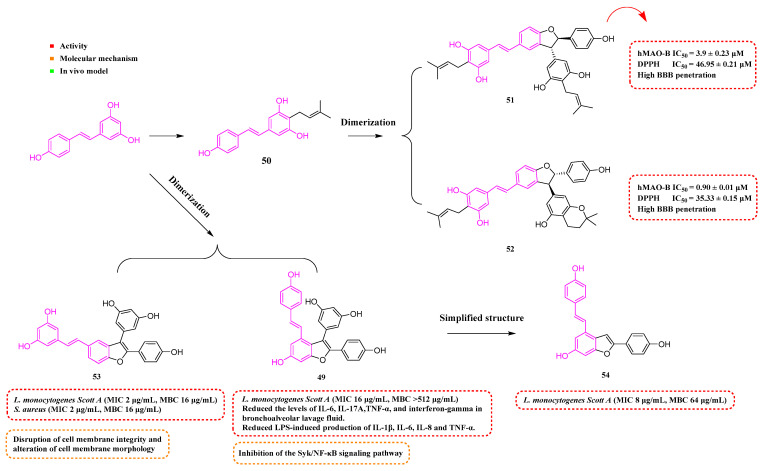
Molecular structure of compounds **49**–**54** and their biological activities.

Fan et al. (2019) uncovered an RSV dimer derivative, amurensin H (**49**, Figure 7), exhibiting anti-inflammatory and antioxidant effects. The investigation revealed that **49** lessened inflammatory markers in lung tissues and reduced the levels of IL-6, IL-17A, TNF-α, and interferon-gamma in bronchoalveolar lavage fluid. It also substantially curtailed LPS-induced production of IL-1β, IL-6, IL-8, and TNF-α. Furthermore, **49** significantly downregulated the expression of p-Syk, NF-κB, and p-NF-κB in laboratory and animal models. Compound **49** also lessened airway inflammation in chronic obstructive pulmonary disease (COPD) [90].

Tang et al. (2019) utilized RSV as a starting material and incorporated an isoprene molecule to synthesize Compound **50** (Figure 7). This compound was then subjected to a dimerization process, resulting in a range of compounds. Notably, Compounds **51** and **52** (Figure 7) exhibited potent inhibitory effects against human monoamine oxidase B (hMAO-B), with IC_50_ values of 3.91 ± 0.23 µM and 0.90 ± 0.01 µM, respectively. Additionally, Compounds **51** and **52** showcased superior antioxidant capabilities and low toxicity in cellular tests, while also exhibiting resistance to neurotoxicity caused by oxidative toxins such as I202, rotenone, and oligomycin-A. Compounds **51** and **52** demonstrated substantial BBB permeability. In addition, these compounds exhibited notable anti-inflammatory effects in vitro, suppressed ROS generation, mitigated apoptosis triggered by H_2_O_2_, and efficiently hindered the release of inflammatory mediators induced by LPS from BV2 cells [91].

Among the monomeric and dimeric RSV derivatives synthesized by Mattio et al. (2019), **49** and **53** (Figure 7) showed considerable antibacterial efficacy against Gram-positive bacteria. The most potent (**53**) showed significant antibacterial activity against LM (MIC = 2 μg/mL, MBC = 16 μg/mL). At a dosage of 100 μg/mL, **53** was able to cause severe damage to cell membranes, including loss of cell membrane potential, impairment of membrane integrity, and severe morphological changes. In addition, it was able to induce cell death in *Staphylococcus aureus* (*S. aureus*) cells at a lower concentration (16 μg/mL) [92]. Catinella et al. (2020) created a streamlined version of Compound **49**, referred to as Compound **54**. This derivative exhibited improved antimicrobial effects against LM, with an MIC of 8 µg/mL and an MBC of 64 µg/mL [93].

## 7. The Key Structural Features and Biological Activities of RSV Derivatives

Structural modifications of RSV derivatives significantly affect their biological activities. By means of different modifications of RSV, such as phenolic hydroxyl groups, benzene rings, linkages, and the introduction of heterocycles, RSV derivatives can be targeted to improve their performance in many key bioactivities, such as anti-inflammatory, anticancer, antioxidant, antimicrobial, and neuroprotective effects.

For example, PTS, a dimethoxy derivative of RSV, has a greatly enhanced lipid solubility, which makes it easier to cross the blood–brain barrier, thus exhibiting stronger antioxidant, anti-inflammatory, and anticancer activities. The stability and water solubility of RSV can be improved by introducing ester groups or long chains through esterification or etherification reactions. For example, certain esterified or etherified derivatives excel in antimicrobial activity, and these compounds have strong inhibitory effects on MRSA, xoo, xoo, xac, etc., by interfering with the integrity of bacterial membranes [30,32,33,34,35]. When RSV binds to glucose or its analogs, it can then cross the blood–brain barrier using GLUT1, enhancing its neuroprotective effects [38,39].

The introduction of heterocyclic structures such as 1,2,3-triazole and 1,3,4-oxadiazole on the benzene ring enhances the antimicrobial activity of the derivatives. For example, derivatives containing the 1,2,3-triazole structure exhibit good inhibition of MRSA [51,52,53,54]. The introduction of double or triple bonds at specific positions of the benzene ring can change the electron distribution and spatial conformation of the derivatives, thus affecting their biological activity. Certain derivatives containing a nitrovinyl structure have excellent anti-inflammatory activity and can precisely target NLRP3 [56,57,58,59].

Replacing the C=C double bond in RSV with a C=N or N=N bond can alter the biological activity of the derivatives. For example, certain RSV derivatives containing C=N bonds excel in antitumor activity [76]. Replacing the C=C double bond with a more flexible amide or single carbon–carbon bond can improve the bioavailability and activity of the derivative. For example, some derivatives containing amide bonds excel in antioxidant and anti-inflammatory activities [79,80].

The introduction of quinoline-containing groups into RSV derivatives excels in anti-inflammatory, antioxidant, and neuroprotective activities. For example, certain quinoline derivatives significantly attenuate brain damage caused by ischemic stroke. Derivatives that introduce an indole group excel in anti-inflammatory and anticancer activities [82,83,84,85]. For example, certain indolyl derivatives significantly inhibit the proliferation and migration of tumor cells [86].

Dimerized derivatives of RSV excel in a variety of biological activities, including antioxidant, anti-inflammatory, antibacterial, and anticancer activities, as well as having good blood–brain barrier permeability [90,91,92,93].

Overall, the parent structure of RSV itself is rich in biological activities. Derivatives with the same or similar structure may show good biological activities in different activity tests. The structural modification of RSV derivatives provides the possibility of their conversion between different biological activities. Through rational design and modification, new RSV derivatives with stronger bioactivity and better pharmacokinetic properties can be developed, providing new strategies and means for the treatment of related diseases.

## 8. Conclusions

RSV is a naturally occurring phenolic stilbene molecule that has been extensively studied for its multiple biological activities, including anti-inflammatory, anticancer, antioxidant, antimicrobial, and neuroprotective properties. However, its clinical application has been hampered by its low water solubility, low bioavailability, and potential hepatotoxicity. In the past five years, significant progress has been made in the structural modification of RSV, resulting in the development of a number of derivatives with enhanced bioactivity and better pharmacological properties. These advances have not only enriched the conformational relationships of RSV derivatives but also deepened our understanding of the drug design and pharmacological activities of RSV derivatives.

Future research should focus on developing RSV derivatives that are more selective for specific targets. For example, derivatives that selectively target the NLRP3 inflammasome could be very effective in treating IBD without causing systemic side effects. Compound **27**, which significantly reduces the release of the inflammatory cytokine IL-1β without triggering the NF-κB pathway, is a promising lead in this direction [59]. The development of RSV derivatives with higher selectivity for cancer cells, especially those expressing specific biomarkers, could enhance their anticancer efficacy. For example, Compound **37** selectively targets cells expressing the CYP1 enzyme, showing potential for the development of more selective anticancer agents [69]. Combining RSV derivatives with other drugs can enhance their therapeutic effects. For example, RSV derivatives with antimicrobial properties can be used in combination with conventional antibiotics against drug-resistant bacteria. Compounds **8** and **9** synergize with aminoglycosides and polymyxin B, demonstrating the potential for combination therapy [40]. The development of RSV derivatives that target multiple pathways in the disease could provide a more comprehensive therapeutic effect. For example, compounds that simultaneously target inflammation, oxidative stress, and cell proliferation may be highly effective in treating complex diseases such as cancer and IBD.

In terms of strategies to improve bioavailability, prodrug strategies can be used to improve the bioavailability of RSV derivatives. For example, synthesizing RSV derivatives with prodrug molecules that can be cleaved in vivo to release active compounds can improve solubility and absorption. This approach has been successfully used in the development of other drugs and can be applied to RSV derivatives as well. Nanoparticle-based drug delivery systems can protect RSV derivatives from degradation and enhance delivery to target tissues. Encapsulation of RSV derivatives in liposomal or polymeric nanoparticles has been investigated to significantly improve their bioavailability and efficacy. For example, RSV derivatives encapsulated in nanoparticles showed increased stability and enhanced delivery to the brain [38].

RSV derivatives are understudied in some respects, and although some RSV derivatives have neuroprotective properties, further research is needed to investigate their mechanisms of action and potential applications in neurodegenerative diseases such as Alzheimer’s and Parkinson’s. Given the current global health challenges, exploring the antiviral activity of RSV derivatives may be a valuable direction of research. RSV has cardiovascular benefits, but more research is needed to develop derivatives specifically for cardiovascular disease. For example, Compound **20** reduces inflammation and oxidative stress and could be further investigated for its potential in the prevention and treatment of cardiovascular disease [51].

High-intensity screening and computational methods can accelerate the discovery of new RSV derivatives. These techniques can rapidly identify promising candidate compounds and predict their biological activity, thereby reducing the time and cost of drug development. For example, Compound **25** was identified as a potent inhibitor of the NLRP3 inflammasome through a high-intensity screening model [56].

Significant progress has been made in the development of new derivatives with enhanced biological activity and better pharmacological properties by structural modification of RSV. These derivatives have shown better anti-inflammatory, anticancer, antimicrobial, antioxidant, and neuroprotective activities, laying a solid foundation for further research and clinical applications. Future research should focus on developing more selective derivatives, improving bioavailability, exploring understudied bioactivities, and employing combination therapies and high-throughput screening methods. These efforts will not only expand the therapeutic potential of RSV derivatives but also contribute to the development of effective new therapies for the treatment of various diseases.

This review collates novel RSV derivatives and their various bioactivities over the past five years and outlines potential future directions, informing further research on the structural modification of RSV and unlocking the therapeutic potential of RSV derivatives.

## Data Availability

Not applicable.

## Abbreviations

The following abbreviations are used in this manuscript:

SARs	Structure–activity relationships
RSV	Resveratrol
PTS	Pterostilbene
BBB	Blood–brain barrier
MRSA	Methicillin-resistant *Staphylococcus aureus*
MIC	Minimum inhibitory concentration
MBC	Minimum bactericidal concentration
EC_50_	Exhibited median effective concentration
Xoo	*Xanthomonas oryzae pv. oryzae*
Xoc	*Xanthomonas oryzae pv. oryzicola*
EPSs	Extracellular polysaccharides
MAPK	Mitogen-activated protein kinase
AD	Atopic dermatitis
DNCB	Dinitrochlorobenzene
GLUT1	Glucose transporter protein 1
ROS	Reactive oxygen species
EAE	Experimental autoimmune encephalomyelitis
LPS	Lipopolysaccharide
HFD	High-fat diet
TNF-α	Tumor necrosis factor-α
IL-6	Interleukin-6
IL-1β	Interleukin-1β
NF-κB	Nuclear transcription factor-κB
COX-2	Cyclooxygenase-2
PA	Palmitic acid
CAT	Catalase
GPX1	Glutathione peroxidase 1
p-ERK1/2	Phosphorylated extracellular signal-regulated kinase 1/2
IR	Inhibition rate
NLRP3	Nucleotide-binding oligomerization domain-like receptor protein 3
IBD	Inflammatory Bowel Disease
HO-1	Oxygenase-1
AChE	Acetylcholinesterase
BChE	Butyrylcholinesterase
PC	Pancreatic cancer
VRE	Vancomycin-resistant *Enterococcus faecium* and *E. faecalis*
CYP1	Cytochrome P450 1
TCDD	Tetrachlorodibenzo-p-dioxin
LM	*Listeria monocytogenes*
HUVECs	Human umbilical vein endothelial cells
SI	Selectivity index
MCAO/R	Middle cerebral artery occlusion–reperfusion
OGD/R	Oxygen–glucose deprivation and reperfusion
IS	Ischemic stroke
COPD	Chronic obstructive pulmonary disease
hMAO-B	Human monoamine oxidase B
STAT3	Transducer and activator of transcription 3
CDK4	Cyclin-dependent kinase 4
